# MicroRNA-181a/b-1 Is Not Required for Innate γδ NKT Effector Cell Development

**DOI:** 10.1371/journal.pone.0145010

**Published:** 2015-12-16

**Authors:** Inga Sandrock, Natalia Ziętara, Marcin Łyszkiewicz, Linda Oberdörfer, Katrin Witzlau, Andreas Krueger, Immo Prinz

**Affiliations:** Institute of Immunology, Hannover Medical School, 30625 Hannover, Germany; University of Tokyo, JAPAN

## Abstract

Thymic development of αβ T lymphocytes into invariant natural killer (NK) T cells depends on their selection via agonistic lipid antigen presented by CD1d. If successful, newly selected NKT cells gain effector functions already in the thymus. Some γδ T cell subsets also acquire effector functions in the thymus. However, it is not clear whether agonistic TCR stimulation is involved in thymic γδ T cell selection and development. Here we combine two genetic models to address this question. MiR-181a/b-1^–/–^mice, which show impaired agonistic T cell selection of invariant αβ NKT cells, were crossed to *Tcrd-H2BeGFP* reporter mice to monitor selection, intra-thymic expansion and differentiation of γδ T cells. We found that miR-181a/b-1-deficiency had no effect on numbers of thymic γδ T cell or on their differentiation towards an IL-17- or IFN-γ-producing effector phenotype. Also, the composition of peripheral lymph node γδ T cells was not affected by miR-181a/b-1-deficiency. Dendritic epidermal γδ T cells were normally present in knock-out animals. However, we observed elevated frequencies and numbers of γδ NKT cells in the liver, possibly because γδ NKT cells can expand and replace missing αβ NKT cells in peripheral niches. In summary, we investigated the role of miR-181a/b-1 for selection, intrathymic development and homeostasis of γδ T cells. We conclude that miR-181a/b-1-dependent modulation of T cell selection is not critically required for innate development of γδ NKT cells or of any other γδ T cell subtypes.

## Introduction

γδ T cells, like αβ T cells, rearrange clonal T cell receptors (TCRs) while they develop in the thymus. Strong evolutionary conservation of γδ T cells in all jawed vertebrates suggests that these cells are essential for immune homeostasis and host competence against infections [1]. In contrast to αβ T cells, the impact of antigen-specific selection of clonal γδ TCR heterodimers is less clear. There is probably no negative selection of thymocytes carrying “wrong” or self-reactive γδ TCRs. However, substantial experimental evidence supports the hypothesis that quality control selection at the DN2—DN3 stage of thymocyte development warrants signaling-competence of γδ TCR heterodimers [2–5]. The necessity of γδ TCR signaling may differ between developing and mature effector γδ T cells, and thus it was suggested that γδ T cells straddle innate and adaptive immunity [6]. According to the signal strength hypothesis, strong signals via the γδ TCR will drive immature thymocytes into the γδ T cell lineage [7–12]. Within that lineage, not all γδ T cells are similar but rather constitute several different subsets that can be grouped according to Vγ-chain-usage and effector phenotype [13, 14]. These subsets develop in progressive waves [14, 15]. Thereby, Vγ5^+^ dendritic epidermal γδ T cells (DETCs) [16, 17] and Vγ6^+^ γδ T cells [18] develop only in the fetal thymus before birth and later persist as self-renewing tissue-resident effector cells. Other tissue-specific γδ T cell populations, including intraepithelial intestinal γδ T cells develop throughout adulthood [19, 20]. Intraepithelial intestinal γδ T cells express TCRs mainly composed of Vγ7 and preferentially pair with Vδ4, Vδ5 and Vδ6 chains [21]. To date, the sole established positive thymic γδ T cell selection was reported for DETCs, which require some specific selecting signal via their invariant Vγ5^+^ Vδ1^+^ TCR for homing to and populating skin epidermis [22, 23]. Furthermore, thymic TCR engagement correlates with the differentiation of thymic γδ T cells into CD122^+^ IFN-γ-secreting effector T cells [24]. There, TCR-triggered CCR6^–^CD27^+^CD122^+^ NK1.1^+/–^ γδ T cells are prone to secrete IFN-γ whereas TCR-untriggered γδ T cells with a CCR6^+^CD44^hi^CD27^–^ phenotype are associated with IL-17 expression [24–26]. In contrast, recent evidence suggested that at least a fraction of CCR6^+^CD27^–^CD44^high^ cells received a strong TCR stimulus very early during thymopoiesis as they become TCR hyporesponsive during development [6]. In this context, it was recently proposed that NK1.1^+^ γδ NKT cells and NK1.1^+^ αβ NKT cells exert similar functions and have an overlapping phenotype [27]. Like αβ NKT cells, γδ NKT cells express the NK cell marker NK1.1 and can rapidly produce IL-4 and IFN-γ. A large proportion of NK1.1^+^ γδ NKT cells express a restricted Vγ1^+^Vδ6.3/6.4^+^ TCR repertoire and start to arise around day 16 of embryonic development [14, 28, 29]. The mechanisms responsible for development and potentially selection of γδ NKT cells are still elusive. Current concepts suggest that agonistic TCR-selection might be required for the development of both αβ NKT cells [30, 31] and γδ NKT cells [29, 32, 33].

We and others recently reported that the miR-181a/b-1 cluster is highly expressed during thymocyte development and positively regulates TCR signal strength [31, 34–36]. Its relative abundance increases during consecutive double negative (DN) stages DN1 to DN4 of thymocyte development from approximately 1%, 2%, 8% to 17% of all miRNAs, respectively, and peaks at >45% in the CD4^+^CD8^+^ DP stage [37]. Accordingly, miR-181a/b-1-deficient animals display severely impaired development of invariant αβ NKT cells, which are agonist-selected at the DP stage, although other cellular functions, such as metabolism or Notch signaling have also been proposed to be regulated by miR-181a/b-1 [31, 38, 39]. In this study, we address whether miR-181a/b-1 influenced a potential agonistic selection of thymic γδ T cells and whether it affected their differentiation towards an IL-17- or IFN-γ-producing effector phenotype. Furthermore, we investigate whether miR-181a/b-1 deficiency has an effect on the development of innate effector γδ NKT cells similar to αβ NKT cells. To this end, we crossed *Tcrd-H2BeGFP* reporter mice [4] to a miR-181a/b-1^–/–^deficient strain [31]. Although staining with antibodies directed against the γδ TCR and CD3ε can also identify most bona fide γδ T cells, the combination of these two genetic models allowed us to better monitor the impact of miR-181a/b-1 on selection, intra-thymic expansion and differentiation of γδ T cells.

## Material and Methods

### Mice

TcrdH2BeGFP (C57BL/6-Tcrdc^tm1Mal^/J;[4]) and miR-181a/b-1^–/–^(C57BL/6-Mirc14^tm1.1Ankr^) were already described. They were crossed to obtain miR-181a/b-1^–/–^x TcrdH2BeGFP mice. F1 C57BL/6 wild type mice (WT, CD45.1/CD45.2) were already described [31]. Mice were bred under specific pathogen free conditions in the central animal facility at Hannover Medical School.

### Ethics

All experiments were conducted according to local and institutional guidelines approved by Lower Saxony State Office for Consumer Protection and Food Safety, file references 11/0533 and 12/0869.

### Flow Cytometry

Antibodies directed against Vγ1 (clone 2.11, PE, 1:100), IFN-γ (clone XMG1.2, PE, 1:100), CD27 (clone LG.3A10, PerCPCy5.5, 1:200), anti-CD117 (clone ACK2, PE, 1:200), anti-CD25 (clone PC61, PerCP-Cy5.5, 1:200), anti-CD45.1 (clone A20, FITC, 1:200) and Tcrβ (H57-597, PECy7, 1:200) were purchased from Biolegend. Anti-CD45.2 (clone 104–2, PerCP-Vio700, 1:200) was purchased from Miltenyi Biotec. Anti-CD24 (clone M1/69, PE, 1:300), CCR6 (clone 11A9, Alexa647, 1:100) and Vδ6.3/2 (clone 8F4H7B7, PE, 1:200) were from BD Biosciences. Antibodies directed against IgM (clone RM-7B4, PE, 1:100), CD44 (clone IM7, eFluor450, 1:200), NK1.1 (clone PK136, PECy7, 1:200), Tcrβ (clone H57-597, APCAlexa750, 1:200) and IL17A (eBio17B7, Alexa647, 1:100) were purchased from eBioscience. Anti-Vγ4 (clone 49.2, subclone49.2–9), subsequently labeled with Cy5, was kindly provided by Pablo Pereira, Paris. 17D1 antibody (rat IgM), here used for staining of Vγ6^+^ γδ T cells, was kindly provided by Bob Tigelaar, Yale. In the presence of unlabeled TCRγδ (GL3) antibody, 17D1 antibodies also recognize Vγ6^+^ γδ T cells in addition to Vγ5^+^ γδ T cells [40]. Binding of 17D1 antibody was detected by anti-rat IgM-PE. APC-conjugated CD1d/PBS-57 (αGalCer analog) loaded and unloaded tetramers were provided by the National Institutes of Health Tetramer Facility at Emory University (Atlanta, GA). Before antibody staining Fc-receptors were blocked with FcR antibody (clone 2.4G2) on ice for 5min. For intracellular staining, cells were stimulated 3h with Phorbol-12-myristate-13-acetate (PMA, 50 ng/ml, Calbiochem) and ionomycin (2 μg/ml, Invitrogen) in the presence of Brefeldin A (1 μg/ml, Sigma). Cells were fixed using the BD Cytofix/ Cytoperm Kit as described in the supplier’s manual. The CD1d/α-GalCer tetramer staining was performed at room temperature for 30min followed by surface staining for 30min on ice. FACS analysis was performed using a LSRII flow cytometer (BD Biosciences) equipped with the BD FACSDiva software. For expression analyses, DN thymocytes enriched by complement lysis were sorted on a FACS Aria II cell sorter (BD) into the following subsets: DN1, CD44^hi^CD117^hi^CD25^–^; DN2, CD44^hi^CD117^hi^CD25^+^; DN3, CD44^–^CD117^–^CD25^+^; DP, CD4^+^CD8^+^; γδ T cells, CD25^–^GFP^+^. Data were analyzed using FlowJo software 9.3.1 (Treestar).

### Cell preparations

Thymocytes were prepared by complement lyses (LowTox-M Rabbit Complement, Cedarlane Laboratories) with anti-CD8 IgM (RL1.72), anti-CD4 IgM (M31) and 300μg DNAseI (Roche). CD4^–^CD8^–^ thymocytes were separated by density gradient using Lympholyte M (Cedarlane laboratories). For the isolation of liver lymphocytes the organs were perfused to remove residual blood. The whole organs were cut into pieces and digested with 0.5mg/ml Collagenase D and 0.025mg/ml DNAseI. Digest was stopped by adding EDTA to a final concentration of 20mM. Digested organs were meshed through a 40μm Cellstrainer (Fisher Scientific). For isolation of lymphocytes from skin, mice were killed and backs of mice were shaved and subsequently treated with depilatory cream (Veet) to remove residual fur. Skin was cut into small pieces and incubated in RPMI supplemented with 1mg/ml Liberase (Roche) and 0,125mg/ml DNAseI for 60min at 37°C, shaking. During the last 15min, EDTA was added to a final concentration of 60mM. Digested skin was meshed through a 100μM Cellstrainer (Fisher Scientific). Liver and skin lymphocytes were separated by density gradient centrifugation using Percoll-gradients.

### Preparation of epidermal sheets

Mouse ears were shaved and dorsal and ventral halves were separated. Both were incubated in 0.5 M NH4SCN at 37°C for 20 min. Epidermal layers were peeled off and were fixed in 4% PFA at RT for 15 min, then rehydrated in TBS-T or PBS for 20 min at RT. Blocking was done with 8% rat serum for 30min at RT. The epidermal sheets were stained with CD3-Cy3 (clone 17A2) at RT for 2 hours and analyzed with an Olympus BX641 microscope. DETCs were identified as GFP^+^CD3^+^.

### MicroRNA Quantitative RT-PCR

RNA preparation and quantitative PCR was performed as described [31] using the RNeasy Mini Kit (Qiagen), TaqMan MicroRNA Reverse Transcription Kit (Applied Biosystems) and miR181a specific primers (TaqMan assay 000480) according to the manufacturers’ protocols. Quantitative RT-PCR analysis of miRNA expression was carried out using the following TaqMan probes: hsa-miR-181a, TM:000480 (Applied Biosystems); hsa-miR-181d, TM:001099 (Applied Biosystems). Relative Expression was calculated as % expression of snoRNA412 as house-keeping microRNA (miRNA) using the ΔCt (Applied Biosystems, TM: 001243).

### Mixed bone marrow chimeras

Bone marrow was isolated from miR-181a/b-1^–/–^(KO, CD45.2) and C57BL/6 wild type (WT, CD45.1/CD45.2) mice. Recipient C57BL/6 wild type (CD45.1) were lethally irradiated (9Gy) and reconstituted intravenously with 10x10^6^ bone marrow cells in a 1:1 mixture of KO (CD45.2) and WT (CD45.1/CD45.2) cells within 4 hours after irradiation. Recipient mice were treated with Cotrimoxazol during the initial two weeks. Analysis was performed after 7 weeks.

### Statistics

Statistical analyses were performed with GraphPad Prism (Version 4.03) using MannWhitney test. P-values <0.05 (*) were considered as significantly different.

## Results

### Thymic development of γδ T cells in the absence of miR-181a/b-1

**Fig 1:**
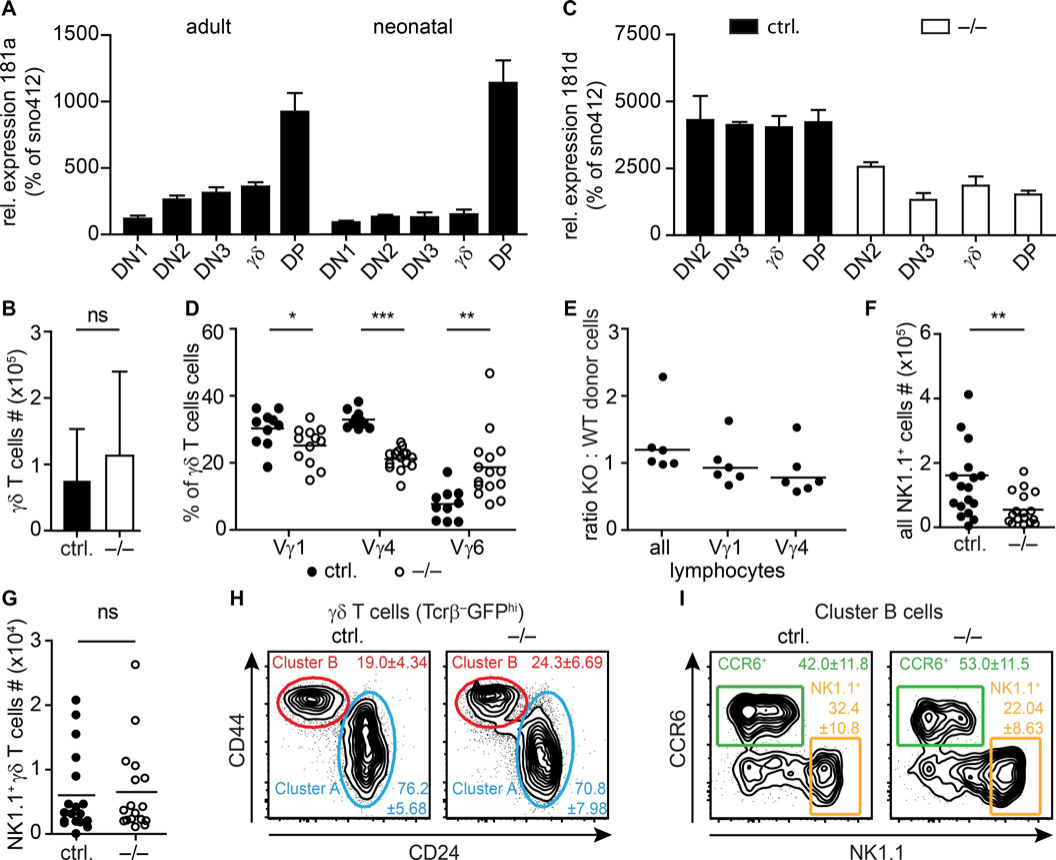
Thymic γδ T cells in the absence of miR-181a/b-1. (A) Expression analysis of miR-181a in FACS-sorted thymocytes pooled from 5 adult or 8 neonatal TcrdH2BeGFP mice. Expression levels of the indicated cell populations were analyzed by quantitative RT-PCR and normalized to snoRNA 412. Error bars show range of relative expression levels from triplicates. (B) Bar graph shows absolute γδ T cell numbers in miR-181a/b-1^–/–^x TcrdH2BeGFP mice (–/–) compared to TcrdH2BeGFP and miR-181a/b-1^+/–^x TcrdH2BeGFP controls (ctrl.), pooled data from five independent experiments with each 2–5 mice per group, mean + SD. (C) Expression analysis of miR-181d in FACS-sorted thymocytes pooled from 5 miR-181a/b-1^–/–^x TcrdH2BeGFP mice (–/–) and TcrdH2BeGFP controls (ctrl.). One representative experiment of two independent experiments that gave similar results. Expression levels of the indicated cell populations were analyzed by quantitative RT-PCR and normalized to snoRNA 412. Error bars show range of relative expression levels from triplicates. (D–I) FACS analysis of thymic γδ T cells in–/–mice compared to ctrl mice (D, F-I) and mixed bone marrow chimeras (E). (D) Vγ usage of thymic γδ T cells (gated on Tcrβ^–^GFP^hi^ cells). Scatter plot shows pooled data from five experiments with 3–6 mice per group, one dot represents one mouse, mean. (E) Flow cytometric analysis of 1:1 mixed bone marrow chimeras. Scatter plot shows ratios of miR-181a/b-1^–/–^(KO) and miR-181a/b-1 sufficient wild type (WT) donor Vγ1^+^ and Vγ4^+^ cells among all lymphocytes, respectively. Data are pooled from two independent experiments with each 3 mice per group, harmonic mean. (F) Scatter plot shows absolute numbers of NK1.1^+^ cells, pooled data from five independent experiments with each 2–5 mice per group. (G) Scatter plot shows absolute numbers of NK1.1^+^ γδ T cells, pooled data from five independent experiments with each 2–5 mice per group. (H) Representative contour plots of cluster B (CD44^hi^CD24^–^) and cluster A (CD44^–/lo^CD24^+^) γδ thymocytes (gated on Tcrβ^–^GFP^hi^ cells), numbers indicate mean +/–SD of pooled data from four independent experiments with each 2–5 mice per group. (I) Representative contour plots of CCR6^+^ and NK1.1^+^ cluster B cells, numbers indicate mean +/–SD of pooled data from four independent experiments with each 2–6 mice per group. Statistical analyses were performed using the Mann-Whitney test.

So far, the role of miR-181 family members in development and selection of γδ T cells was not investigated in detail. The miR-181a/b-1 cluster is differentially expressed across all DN immature stages of thymocyte development. It peaks with the highest copy number per cell and the highest relative expression of all miRNAs in DP thymocytes [36]. However, RNA-sequencing revealed that miR-181a was already the fifteenth and third most abundant of all miRNAs in the DN2 and DN3 stages, respectively [37]. The late DN2 to DN3 developmental stages frame the window, in which γδ T cells branch off to further develop as a separate T cell lineage [9, 10]. In order to confirm the high abundance of miR-181a/b-1 in immature thymocytes, we measured expression of miR-181a in FACS-sorted thymocyte subsets derived from TcrdH2BeGFP mice. Here we found comparably high expression levels of miR-181a in DN2, DN3, and γδ thymocytes as shown by relative quantification of miR-181a in comparison to the small nuclear RNA sno412 (Fig 1A). Thus, our data essentially reproduced previously published miRNA expression analyses performed by RNA sequencing [37]. Notably, similar expression dynamics were observed in thymocytes sorted from neonatal thymi, albeit absolute expression levels in DN and γδ thymocytes were lower as compared to adult thymi.

In miR-181a/b-1^–/–^mice the development of αβ NKT cells and most prominently that of CD1d-restricted semi-invariant so-called iNKT cells, is disturbed as indicated by massively reduced numbers of αβ KT cells [31, 39]. To address if deletion of miR-181a/b-1 affected thymic γδ T cell numbers, we compared numbers of thymic γδ T cells of miR-181a/b-1 deficient mice to miR-181a/b-1 sufficient littermate controls. However, γδ T cells numbers were not significantly different in the absence of miR-181a/b-1 (Fig 1B). To evaluate a possible compensatory up regulation of other members of the miR181family that might compensate the lack of 181a/b-1, we analyzed the expression of miR181d in WT compared to 181KO mice in sorted thymocytes. Relative quantification revealed no compensatory up-regulation of miR181d in miR-181a/b-1 knockout thymocytes (Fig 1C). These data are consistent with previous studies showing that miR-181a and miR-181b are predominantly expressed from mir-181ab1 in thymocytes [38]. As miR-181a/b-1 was shown to influence the TCR repertoire of iNKT cells [31], we next investigated the Vγ-chain usage of thymic γδ T cells. Interestingly, the frequency of Vγ1^+^ and Vγ4^+^ γδ T cells was lower in miR-181a/b-1^–/–^mice, whereas the frequency of Vγ6^+^ γδ T cells was increased compared to miR-181a/b-1-sufficient controls (Fig 1D). Such a shift in Vγ-chain usage towards Vγ6^+^ γδ T cells might reflect that development and thymic persistence of the latter are relatively less dependent on miR-181a/b-1-modulated TCR signal sensitivity. To address a possible cell intrinsic advantage or disadvantage of miR181a/b-1 deficient compared to control thymocytes we analyzed the Vγ1^+^ and Vγ4^+^ thymocytes in mixed bone marrow chimeras. Therefore, lethally irradiated wild type mice were reconstituted with a 1:1 mixture of miR-181a/b-1 knockout and wild type bone marrow cells. The ratios of miR-181a/b-1^–/–^donor cells and wild type donor cells, identified by congenic markers, were approximately one, indicating that none of the donor cells had a cell intrinsic advantage over the other (Fig 1E).

The cumulative number of NK1.1^+^ T cells in thymus was suggested to be tightly controlled by competition for thymic NKT cell niches [27]. Therefore, we assessed the number of total NK1.1^+^ cells and NK1.1^+^ γδ T cells. While the number of all NK1.1^+^ cells was reduced in miR-181a/b-1^–/–^mice due to impaired development of αβ iNKT cells (Fig 1F), numbers of NK1.1^+^ γδ T cells were comparable to miR-181a/b-1-sufficient controls (Fig 1G). This suggests that, although expanded populations of NK1.1^+^ γδ T cells compromised the number of thymic αβ iNKT cells in TCR γ-chain-transgenic mice [27], reduced numbers of αβ iNKT cells did not *vice versa* lead to a thymic niche-dependent expansion of NK1.1^+^ γδ T cells. Next, we investigated whether intrathymic development into CD24^–^CD44^hi^ cluster B [4] effector γδ T cells was affected by miR-181a/b-1-deficiency. We found no alterations in the frequency of cluster B cells (Fig 1H). Within cluster B proportions of CCR6^+^ and NK1.1^+^ effector γδ T cells (Fig 1I), which mark IL-17- or IFN-γ- producing effector γδ T cells, respectively, were also unaltered. Together, this indicates that deletion of miR-181a/b-1 does neither alter thymic γδ NKT cells numbers nor their thymic development into cluster B effector T cells.

### Unchanged peripheral lymph node γδ T cells in the absence of miR-181a/b-1

**Fig 2:**
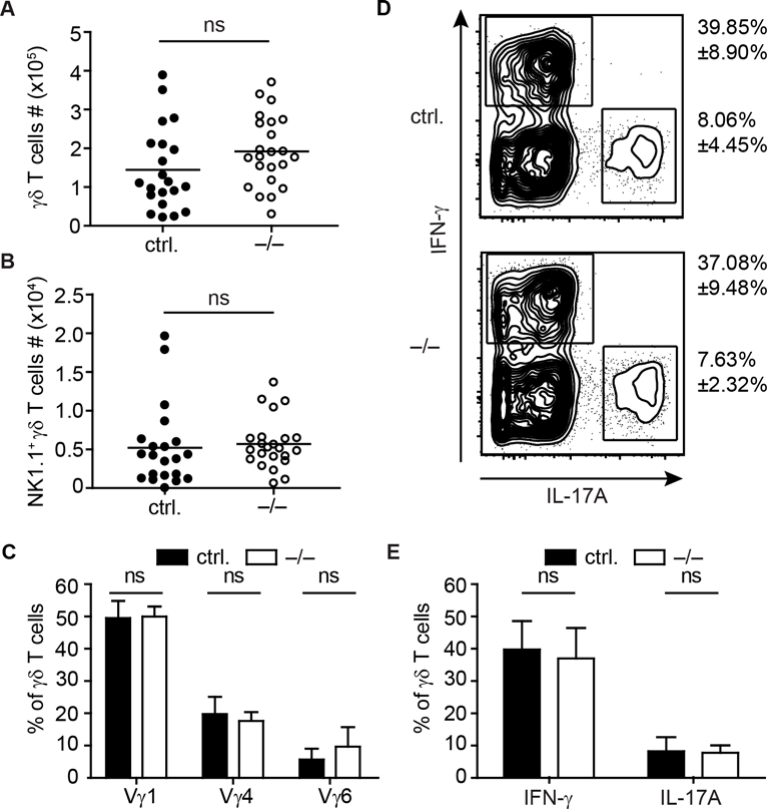
Unchanged peripheral lymph node γδ T cell compartment in the absence of miR-181a/b-1. FACS analysis of γδ T cells in pLN of miR-181a/b-1^–/–^x TcrdH2BeGFP mice (–/–) compared to miR-181a/b-1 sufficient controls, TcrdH2BeGFP and miR-181a/b-1^+/–^x TcrdH2BeGFP mice (here referred to as ctrl.). (A) Total γδ T cells numbers in pLN of the indicated phenotypes. Scatter plot shows pooled data from five independent experiment with n = 2–5 mice per group, mean. (B) Scatter plot shows absolute numbers of NK1.1^+^ γδ T cells, pooled data from five independent experiments with each 2–5 mice per group, mean. (C) Vγ usage of γδ T cells (gated on Tcrβ^–^GFP^hi^ cells). Bar graph shows pooled data from 5 experiments with 3–6 mice per group, mean + SD. (D + E) Intracellular cytokine staining for IFN-γ and IL-17A gated on γδ T cells. (D) Representative contour plots of two independent experiments with similar outcome, with each n = 2–5 mice per group. Numbers indicate mean +/–SD from pooled data. (E) Bar gaph shows pooled data from the two independent experiments, mean + SD. Statistical analyses were performed using the Mann-Whitney test.

In miR-181a/b-1^–/–^mice the paucity of iNKT cells is not restricted to the thymic compartment but also transmitted into periphery [31]. Peripheral lymph nodes (pLN) displayed no difference of total γδ T cell numbers in miR-181a/b-1^–/–^mice and miR-181a/b-1 sufficient controls (Fig 2A). In addition, similar to the thymus, numbers of NK1.1^+^ γδ T cells in pLN were comparable (Fig 2B). As we detected a moderate but significantly altered Vγ chain usage in thymi of miR-181a/b-1^–/–^mice (Fig 1C), we next investigated the peripheral γδ TCR repertoire. To this end, we analyzed Vγ chain usage via antibody staining for Vγ1, Vγ4 and Vγ6 chains. However, we did not observe differences between miR-181a/b-1^–/–^mice and controls in pLN γδ T cells (Fig 2C). Furthermore, pLN displayed no difference in IL-17- or IFN-γ- producing effector γδ T cells as revealed by intracellular cytokine staining (Fig 2D and 2E)

### NK1.1^+^ γδ NKT cells fill empty iNKT niches in the liver of miR-181a/b-1-deficient mice

**Fig 3:**
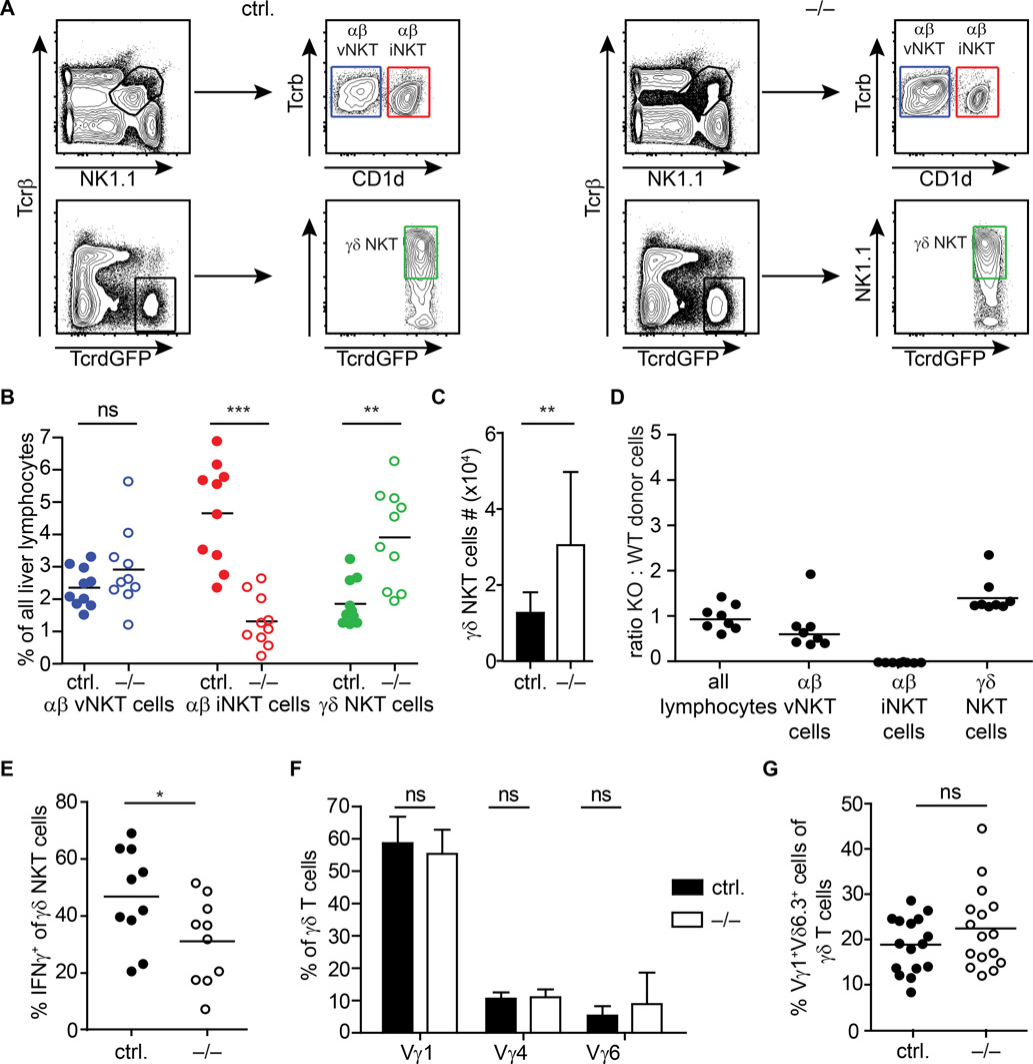
γδ NKT cells fill empty iNKT liver niches in miR-181a/b-1 deficient mice. FACS analysis of liver lymphocytes of miR-181a/b-1^–/–^x TcrdH2BeGFP mice (–/–) compared to TcrdH2BeGFP and miR-181a/b-1^+/–^x TcrdH2BeGFP controls (here referred to as ctrl.). (A + B) Analysis of αβ and γδ NKT cells in miR181a/b-1 deficient mice compared to controls. (A) Representative contour plots illustrating the gating strategy for the indicated cell populations in (B). (B) Scatter plot shows frequencies of the indicated cell populations among lymphocytes after doublets were excluded, gated as depicted in (A), pooled data from three independent experiments with each 3–4 mice per group, mean. (C) Bar graph shows total γδ NKT cell numbers, pooled data from 3 independent experiments, each n = 3–4 mice per group, mean + SD. (D) Flow cytometric analysis of 1:1 mixed bone marrow chimeras. Scatter plot shows ratios of miR-181a/b-1^–/–^(KO) and miR-181a/b-1 sufficient wild type (WT) donor cells among all lymphocytes, αβ vNKT, αβ iNKT and γδ NKT lymphocytes, respectively, pooled data from two independent experiments with each 4 mice per group, harmonic mean. (E) Scatter plot shows frequencies of INFγ^+^ cells among γδ T cells, pooled data from three independent experiments with each 2–5 mice per group, mean. (F) Vγ usage of liver γδ T cells (gated on Tcrβ^–^GFP^hi^ cells). Bar graph shows pooled data from five experiments with 3–6 mice per group, mean + SD. (G) Analysis of liver Vγ1^+^Vδ6.3^+^ γδ T cells. Scatter plot shows frequencies of Vγ1^+^Vδ6.3^+^ cells among γδ T cells, pooled data from five independent experiments with each 2–5 mice per group, mean. Statistical analyses were performed using the Mann-Whitney test.

γδ NKT cells mainly home to and reside within the liver, similar to αβ iNKT cells [41]. Therefore, we analyzed the composition of the γδ T cell compartment in the liver of miR-181a/b-1^–/–^mice. First, we compared the frequencies of γδ NKT cells, here defined as NK1.1^+^ γδ T cells, and αβ NKT cells in miR-181a/b-1 deficient mice compared to sufficient controls. Frequencies as well as numbers of NK1.1^+^ γδ NKT cells were significantly elevated in miR-181a/b-1^–/–^mice compared to controls (Fig 3A–3C). At the same time, numbers and frequencies of liver-resident NK cells and conventional αβ T cells were similar in miR-181a/b-1^–/–^and control mice (data not shown). Analysis of mixed bone marrow chimeras indicated that miR-181a/b-1^–/–^ γδ NKT cells had no cell intrinsic advantage compared to miR-181a/b-1 sufficient γδ NKT cells (Fig 3D). Together, these results suggest that NK1.1^+^ γδ NKT cells populate and expand in niches in the liver that are free due to reduced numbers of αβ iNKT cells. Next, we analyzed the effector functions of NK1.1^+^ γδ T cells by intracellular cytokine staining for IFN-γ. Cells from miR181a/b-1 deficient as well as from sufficient control mice were both capable to produce IFN-γ after *in vitro* stimulation with PMA/ionomycin although the frequency of IFN-γ^+^ γδ NKT cells was slightly reduced in the knockout mice (Fig 3D). Further, we assessed the Vγ4, Vγ6 and Vγ1 chain usage of liver γδ T cells. Similar to the analysis of pLN (Fig 2C), we did not observe any differences in miR-181a/b-1 deficient mice compared to sufficient controls (Fig 3F). A sizable fraction of NK1.1^+^ γδ NKT cells express a semi invariant Vγ1^+^Vδ6.3^+^ TCR [29]. To determine whether the expansion of NK1.1^+^ γδ NKT cells correlates with an expansion of this semi invariant γδ NKT cell subset, we next analyzed the expression of Vγ1 and Vδ6.3 on liver γδ T cells by FACS. Although TCR-specificity plays a critical role in the development of Vγ1^+^Vδ6.3^+^ γδ T cells, their frequencies were unchanged in miR-181a/b-1 deficient as compared to sufficient controls (Fig 3G). Together, this indicates that augmented numbers of NK1.1^+^ γδ NKT cells in the liver of miR-181a/b-1 deficient mice are not due to expansion of semi invariant Vγ1^+^Vδ6.3^+^ γδ T cells, but rather result from increased frequencies of NK1.1^+^ γδ NKT cells using other TCR chain combinations.

### γδ NKT cells replenish empty niches of missing liver αβ iNKT cells independent of TCR specificity for CD1d

**Fig 4:**
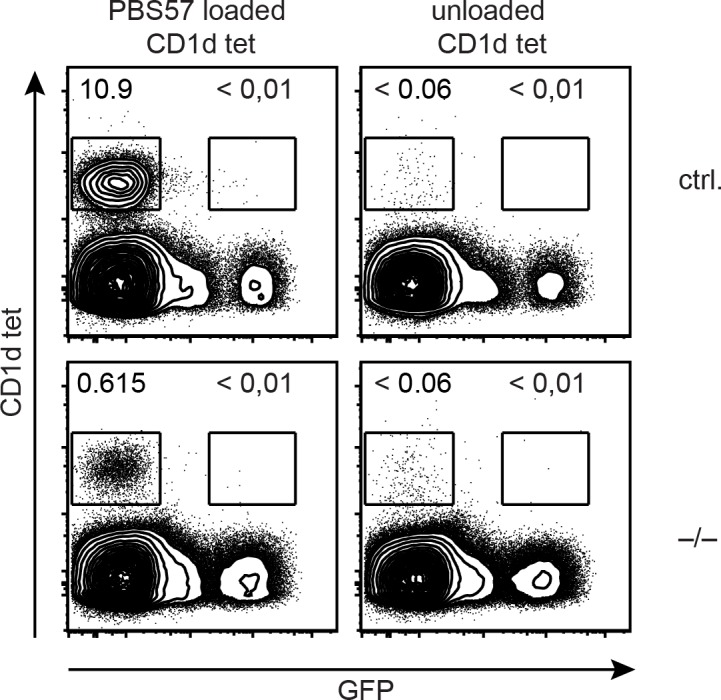
γδ NKT cells replenish empty niches of missing liver αβ iNKT cells independent of TCR specificity for CD1d. FACS analysis of liver lymphocytes stained for CD1d/PBS-57 tetramer (CD1d tet) binding. Contour plots show representative CD1d tet binding versus γδ-GFP reporter fluorescence from two independent experiments, with each involving 3 mice per group of miR-181a/b-1 deficient (–/–) and miR-181a/b-1 sufficient (ctrl.) mice.

Next, we addressed whether the balance between αβ and γδ NKT cells may depend on direct competition for signals transduced via the TCR. iNKT cells are agonist-selected for recognition of lipid-Ag presented by CD1d. If the two cell types competed for the same niche it is conceivable that they share their TCR specificities. To test this, we analyzed whether H2BeGFP^+^ γδ T cells bound CD1d tetramers loaded with the αGalCer analog PBS-57. H2BeGFP^+^ γδ T cells did not bind the tetramer (Fig 4), suggesting that γδ T cells replenish the liver iNKT cell niche independent of CD1d-specific TCR triggering. These data are consistent with previous reports that showed that γδ NKT cells did not bind to CD1d-αGalCer and developed in the absence of CD1d [27, 32, 33].

### Normal DETC numbers and phenotype in the absence of miR-181a/b-1

**Fig 5:**
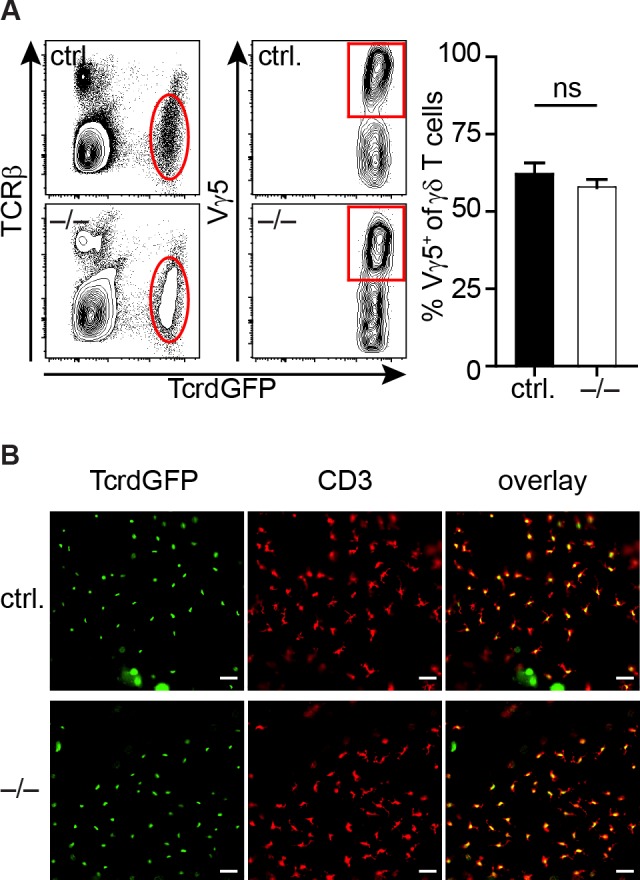
Dendritic epidermal T cells develop in the absence of miR-181a/b-1. (A) Analysis of Vγ5^+^ γδ T cells in the skin of miR-181a/b-1^–/–^x TcrdH2BeGFP mice (–/–) compared to TcrdH2BeGFP mice (ctrl.). (left) Representative contour plots illustrating the gating for Vγ5^+^ γδ T cells. (right) Scatter plot shows frequencies of Vγ5^+^ γδ T cells, pooled data from two independent experiments with each n = 3 mice per group, mean. (B) Histological analysis of epidermal sheets from ears of miR-181a/b-1^–/–^x TcrdH2BeGFP mice (–/–) compared to TcrdH2BeGFP mice (ctrl.). Epidermal sheets were stained for DETCs (yellow, overlay red and green) with CD3 (red) and TcrdGFP^+^ (green) cells indicate γδ T cells. Original magnification: 20x, scale bars: 20μm.

Finally, we tested whether the absence of miR-181a/b-1 would impact the presence of DETCs, which are to date the sole γδ T cell population with an established requirement for TCR-specific positive thymic selection [12, 22, 23, 42]. However, we detected no differences in frequencies or phenotype as shown by FACS analysis of skin lymphocytes (Fig 5A) as well as by histological analysis of epidermal sheets from ears of miR-181a/b-1-deficient and miR-181a/b-1-proficient TcrdH2BeGFP reporter mice (Fig 5B).

## Discussion

The aim of this study was to analyze the impact of miR-181a/b-1-deficiency on the development and homeostasis of γδ T cells. The rationale behind this aim was straightforward: The miR-181a/b-1 cluster is highly expressed during thymocyte development and positively regulates TCR signal strength [31, 35, 36]. Consequently, two recent studies had revealed a critical role for miR-181a/b-1 in development of agonist-selected invariant αβ NKT cells using two independent loss of function knock-out mice lines [31, 39]. Thus, it was likely that miR-181a/b-1-deficiency would also alter the efficiency of γδ T cell production of those subsets that potentially required agonistic TCR signals for their thymic selection.

Nonetheless, overall numbers and frequencies of thymic and peripheral γδ T cells were largely unaltered in the absence of miR-181a/b-1. Analysis of mixed bone marrow chimeras indicated that, in general, the development of γδ thymocytes was not compromised by miR-181a/b-1 deficiency. Both, miR-181a/b-1 deficient as well as wild type donor lymphocytes comparably reconstituted Vγ1^+^ and Vγ4^+^ thymocytes. However, we observed a slight TCR repertoire shift in miR-181a/b-1 deficient thymic γδ T cells. Importantly, these results were stable when heterozygous littermates were used as controls, thereby excluding unrelated effects of the genetic background. In the thymus, we found a higher frequency of thymic Vγ6^+^ cells in miR-181a/b-1-deficient mice. These invariant Vγ6^+^Vδ1^+^ cells are largely contributing to the pool of CCR6^+^ IL-17-producing effector γδ T cells [43–45]. This was not necessarily expected from the literature, since deficiency in miR-181a/b-1 lead to reduced expression of Notch target genes, including. Hes1 [38], and in turn, Hes1-deficient mice had severely decreased numbers of IL-17 producing γδ T cells[46]. Although these results were very reproducible and statistically highly significant, their biological relevance still needs to be determined in future studies. Since such CCR6^+^ IL-17-producing effector γδ T cells were suggested to require no antigen-specific TCR engagement for their differentiation [24, 42], it is conceivable that their thymic development is relatively less affected by the absence of miR-181a/b-1. Interestingly, a recent report showed that IFN-γ-producing and IL-17-producing γδ T cells developed from DN2 cells, while only IFN-γ-producing γδ T cells developed from DN3 cells [47]. Consistent with the dominant expression pattern of miR-181a/b-1 in thymocytes, these findings support the idea that this miRNA is less important at early developmental stages. Still, deficiency of one miR-cluster might have been be compensated by others. Therefore, it would be instructive to investigate knock-out mice deficient for more than one miR-181 cluster. Although mice with a complete knock-out of all three miR-181 clusters are presumably lethal [38, 39]future work might rely on the combined T cell progenitor specific conditional deletion of all threemiR-181 clusters. Overall, this study revealed a mild effect of miR-181a/b-1-deficiency on the γδ TCR repertoire, which is consistent with a moderate expression level of the miR-181a/b-1-cluster in DN3 thymocytes and in γδ T cells.

On the other hand, we found no impairment of two potentially agonist-selected γδ T cell populations, namely DETCs and γδ NKT. For the former, our conclusions are based on microscopic analysis of adult epidermis. However, a thorough analysis of their early thymic ontogeny would be required to make a statement that DETC development and selection are not at all affected by the presence or absence of miR-181a/b-1. For the latter, our data underline that αβ NKT cells and γδ NKT cells share many but not all features. It was previously reported that αβ NKT cells and γδ NKT cells share some developmental requirements and home to similar anatomical sites [41]. While both lineages are controlled by E-proteins [48] and PLZF [27, 28, 33], the influence of TCR engagement for development and expansion of γδ NKT cells is less central [32, 33, 49]. In contrast to previous studies [27], we did not note competition of αβ NKT cells and γδ NKT cells for a thymic niche. Nevertheless, our finding of elevated NK1.1^+^γδ NKT cell frequencies in the liver implies that γδ NKT cells can expand and partially replace missing αβ NKT cells in peripheral niches, confirming that αβ NKT and γδ NKT cells can compete for homeostatic niches. As these two cell types do not compete for (CD1d-restricted) TCR antigens, it will be interesting to identify the factors that define the homeostasis of αβ NKT and γδ NKT cells, but not of NK cells or CD8^+^ αβ T cells, in the liver. Also, given the myriad functions of miR-181a/b-1 in T cell development, our study does not formally exclude a cell intrinsic function of miR-181a/b-1 in γδ NKT cell development and selection.

In conclusion, this study investigated the impact of miR-181a/b-1 on γδ T cell development. We found that miR-181a/b-1-deficiency alters the γδ TCR repertoire to some degree, whereas tuning of TCR sensitivity by the miR-181a/b-1-cluster is not critically required for the development and expansion of γδ NKT cells.

## References

[1] Chien YH, Meyer C, Bonneville M. gammadelta T cells: first line of defense and beyond. Annu Rev Immunol. 2014;32:121–55. Epub 2014/01/07. 10.1146/annurev-immunol-032713-120216 . 24387714

[2] Livak F, Petrie HT, Crispe IN, Schatz DG. In-frame TCR delta gene rearrangements play a critical role in the alpha beta/gamma delta T cell lineage decision. Immunity. 1995;2(6):617–27. Epub 1995/06/01. doi: 1074-7613(95)90006-3 [pii]. . 7796295 10.1016/1074-7613(95)90006-3

[3] Passoni L, Hoffman ES, Kim S, Crompton T, Pao W, Dong MQ, et al Intrathymic delta selection events in gammadelta cell development. Immunity. 1997;7(1):83–95. Epub 1997/07/01. doi: S1074-7613(00)80512-9 [pii]. . 9252122 10.1016/s1074-7613(00)80512-9

[4] Prinz I, Sansoni A, Kissenpfennig A, Ardouin L, Malissen M, Malissen B. Visualization of the earliest steps of gammadelta T cell development in the adult thymus. Nat Immunol. 2006;7(9):995–1003. Epub 2006/08/01. doi: ni1371 [pii] 10.1038/ni1371 . 16878135

[5] Taghon T, Yui MA, Pant R, Diamond RA, Rothenberg EV. Developmental and molecular characterization of emerging beta- and gammadelta-selected pre-T cells in the adult mouse thymus. Immunity. 2006;24(1):53–64. Epub 2006/01/18. doi: S1074-7613(05)00407-3 [pii] 10.1016/j.immuni.2005.11.012 . 16413923

[6] Wencker M, Turchinovich G, Di Marco Barros R, Deban L, Jandke A, Cope A, et al Innate-like T cells straddle innate and adaptive immunity by altering antigen-receptor responsiveness. Nat Immunol. 2014;15(1):80–7. Epub 2013/11/19. doi: ni.2773 [pii] 10.1038/ni.2773 . 24241693 PMC6485477

[7] Haks MC, Lefebvre JM, Lauritsen JP, Carleton M, Rhodes M, Miyazaki T, et al Attenuation of gammadeltaTCR signaling efficiently diverts thymocytes to the alphabeta lineage. Immunity. 2005;22(5):595–606. Epub 2005/05/17. doi: S1074-7613(05)00130-5 [pii] 10.1016/j.immuni.2005.04.003 . 15894277

[8] Hayes SM, Li L, Love PE. TCR signal strength influences alphabeta/gammadelta lineage fate. Immunity. 2005;22(5):583–93. Epub 2005/05/17. doi: S1074-7613(05)00109-3 [pii] 10.1016/j.immuni.2005.03.014 . 15894276

[9] Ciofani M, Knowles GC, Wiest DL, von Boehmer H, Zuniga-Pflucker JC. Stage-specific and differential notch dependency at the alphabeta and gammadelta T lineage bifurcation. Immunity. 2006;25(1):105–16. Epub 2006/07/04. doi: S1074-7613(06)00296-2 [pii] 10.1016/j.immuni.2006.05.010 . 16814577

[10] Garbe AI, Krueger A, Gounari F, Zuniga-Pflucker JC, von Boehmer H. Differential synergy of Notch and T cell receptor signaling determines alphabeta versus gammadelta lineage fate. J Exp Med. 2006;203(6):1579–90. Epub 2006/06/07. doi: jem.20060474 [pii] 10.1084/jem.20060474 16754723 PMC2118312

[11] Kreslavsky T, Garbe AI, Krueger A, von Boehmer H. T cell receptor-instructed alphabeta versus gammadelta lineage commitment revealed by single-cell analysis. J Exp Med. 2008;205(5):1173–86. Epub 2008/04/30. doi: jem.20072425 [pii] 10.1084/jem.20072425 18443226 PMC2373848

[12] Turchinovich G, Pennington DJ. T cell receptor signalling in gammadelta cell development: strength isn't everything. Trends Immunol. 2011;32(12):567–73. Epub 2011/11/08. doi: S1471-4906(11)00153-0 [pii] 10.1016/j.it.2011.09.005 . 22056207

[13] Pang DJ, Neves JF, Sumaria N, Pennington DJ. Understanding the complexity of gammadelta T-cell subsets in mouse and human. Immunology. 2012;136(3):283–90. Epub 2012/03/06. 10.1111/j.1365-2567.2012.03582.x 22385416 PMC3385028

[14] Prinz I, Silva-Santos B, Pennington DJ. Functional development of gammadelta T cells. Eur J Immunol. 2013;43(8):1988–94. Epub 2013/08/10. 10.1002/eji.201343759 . 23928962

[15] Carding SR, Egan PJ. Gammadelta T cells: functional plasticity and heterogeneity. Nat Rev Immunol. 2002;2(5):336–45. Epub 2002/05/30. 10.1038/nri797 . 12033739

[16] Asarnow DM, Kuziel WA, Bonyhadi M, Tigelaar RE, Tucker PW, Allison JP. Limited diversity of gamma delta antigen receptor genes of Thy-1+ dendritic epidermal cells. Cell. 1988;55(5):837–47. Epub 1988/12/02. doi: 0092-8674(88)90139-0 [pii]. . 2847872 10.1016/0092-8674(88)90139-0

[17] Havran WL, Grell S, Duwe G, Kimura J, Wilson A, Kruisbeek AM, et al Limited diversity of T-cell receptor gamma-chain expression of murine Thy-1+ dendritic epidermal cells revealed by V gamma 3-specific monoclonal antibody. Proc Natl Acad Sci U S A. 1989;86(11):4185–9. Epub 1989/06/01. 2726770 10.1073/pnas.86.11.4185 PMC287415

[18] Itohara S, Farr AG, Lafaille JJ, Bonneville M, Takagaki Y, Haas W, et al Homing of a gamma delta thymocyte subset with homogeneous T-cell receptors to mucosal epithelia. Nature. 1990;343(6260):754–7. Epub 1990/02/22. 10.1038/343754a0 . 2154700

[19] Chennupati V, Worbs T, Liu X, Malinarich FH, Schmitz S, Haas JD, et al Intra- and intercompartmental movement of gammadelta T cells: intestinal intraepithelial and peripheral gammadelta T cells represent exclusive nonoverlapping populations with distinct migration characteristics. J Immunol. 2010;185(9):5160–8. Epub 2010/09/28. doi: jimmunol.1001652 [pii] 10.4049/jimmunol.1001652 . 20870939

[20] Haas JD, Ravens S, Duber S, Sandrock I, Oberdorfer L, Kashani E, et al Development of interleukin-17-producing gammadelta T cells is restricted to a functional embryonic wave. Immunity. 2012;37(1):48–59. Epub 2012/07/10. doi: S1074-7613(12)00240-3 [pii] 10.1016/j.immuni.2012.06.003 . 22770884

[21] Grigoriadou K, Boucontet L, Pereira P. T cell receptor-gamma allele-specific selection of V gamma 1/V delta 4 cells in the intestinal epithelium. J Immunol. 2002;169(7):3736–43. Epub 2002/09/24. . 12244167 10.4049/jimmunol.169.7.3736

[22] Xiong N, Kang C, Raulet DH. Positive selection of dendritic epidermal gammadelta T cell precursors in the fetal thymus determines expression of skin-homing receptors. Immunity. 2004;21(1):121–31. Epub 2004/09/04. 10.1016/j.immuni.2004.06.008 S1074761304001633 [pii]. . 15345225

[23] Lewis JM, Girardi M, Roberts SJ, Barbee SD, Hayday AC, Tigelaar RE. Selection of the cutaneous intraepithelial gammadelta+ T cell repertoire by a thymic stromal determinant. Nat Immunol. 2006;7(8):843–50. Epub 2006/07/11. doi: ni1363 [pii] 10.1038/ni1363 . 16829962

[24] Jensen KD, Su X, Shin S, Li L, Youssef S, Yamasaki S, et al Thymic selection determines gammadelta T cell effector fate: antigen-naive cells make interleukin-17 and antigen-experienced cells make interferon gamma. Immunity. 2008;29(1):90–100. Epub 2008/07/01. doi: S1074-7613(08)00271-9 [pii] 10.1016/j.immuni.2008.04.022 18585064 PMC2601709

[25] Haas JD, Gonzalez FH, Schmitz S, Chennupati V, Fohse L, Kremmer E, et al CCR6 and NK1.1 distinguish between IL-17A and IFN-gamma-producing gammadelta effector T cells. Eur J Immunol. 2009;39(12):3488–97. Epub 2009/10/16. 10.1002/eji.200939922 . 19830744

[26] Ribot JC, deBarros A, Pang DJ, Neves JF, Peperzak V, Roberts SJ, et al CD27 is a thymic determinant of the balance between interferon-gamma- and interleukin 17-producing gammadelta T cell subsets. Nat Immunol. 2009;10(4):427–36. Epub 2009/03/10. doi: ni.1717 [pii] 10.1038/ni.1717 19270712 PMC4167721

[27] Pereira P, Boucontet L. Innate NKTgammadelta and NKTalphabeta cells exert similar functions and compete for a thymic niche. Eur J Immunol. 2012;42(5):1272–81. Epub 2012/04/28. 10.1002/eji.201142109 . 22539299

[28] Kreslavsky T, Savage AK, Hobbs R, Gounari F, Bronson R, Pereira P, et al TCR-inducible PLZF transcription factor required for innate phenotype of a subset of gammadelta T cells with restricted TCR diversity. Proc Natl Acad Sci U S A. 2009;106(30):12453–8. Epub 2009/07/21. doi: 0903895106 [pii] 10.1073/pnas.0903895106 19617548 PMC2718370

[29] Pereira P, Berthault C, Burlen-Defranoux O, Boucontet L. Critical role of TCR specificity in the development of Vgamma1Vdelta6.3+ innate NKTgammadelta cells. J Immunol. 2013;191(4):1716–23. Epub 2013/07/16. doi: jimmunol.1203168 [pii] 10.4049/jimmunol.1203168 . 23851687

[30] Stritesky GL, Jameson SC, Hogquist KA. Selection of self-reactive T cells in the thymus. Annu Rev Immunol. 2012;30:95–114. Epub 2011/12/14. 10.1146/annurev-immunol-020711-075035 22149933 PMC3518413

[31] Zietara N, Lyszkiewicz M, Witzlau K, Naumann R, Hurwitz R, Langemeier J, et al Critical role for miR-181a/b-1 in agonist selection of invariant natural killer T cells. Proc Natl Acad Sci U S A. 2013;110(18):7407–12. Epub 2013/04/17. doi: 1221984110 [pii] 10.1073/pnas.1221984110 23589855 PMC3645533

[32] Azuara V, Lembezat MP, Pereira P. The homogeneity of the TCRdelta repertoire expressed by the Thy-1dull gammadelta T cell population is due to cellular selection. Eur J Immunol. 1998;28(11):3456–67. Epub 1998/12/08. 10.1002/(SICI)1521-4141(199811)28:11<3456::AID-IMMU3456>3.0.CO;2-F [pii]. . 9842888

[33] Alonzo ES, Gottschalk RA, Das J, Egawa T, Hobbs RM, Pandolfi PP, et al Development of promyelocytic zinc finger and ThPOK-expressing innate gamma delta T cells is controlled by strength of TCR signaling and Id3. J Immunol. 2010;184(3):1268–79. Epub 2009/12/30. doi: jimmunol.0903218 [pii] 10.4049/jimmunol.0903218 20038637 PMC3991695

[34] Chen CZ, Li L, Lodish HF, Bartel DP. MicroRNAs modulate hematopoietic lineage differentiation. Science. 2004;303(5654):83–6. Epub 2003/12/06. 10.1126/science.1091903 [pii]. . 14657504

[35] Li QJ, Chau J, Ebert PJ, Sylvester G, Min H, Liu G, et al miR-181a is an intrinsic modulator of T cell sensitivity and selection. Cell. 2007;129(1):147–61. Epub 2007/03/27. doi: S0092-8674(07)00319-4 [pii] 10.1016/j.cell.2007.03.008 . 17382377

[36] Neilson JR, Zheng GX, Burge CB, Sharp PA. Dynamic regulation of miRNA expression in ordered stages of cellular development. Genes Dev. 2007;21(5):578–89. Epub 2007/03/09. doi: 21/5/578 [pii] 10.1101/gad.1522907 17344418 PMC1820899

[37] Kirigin FF, Lindstedt K, Sellars M, Ciofani M, Low SL, Jones L, et al Dynamic microRNA gene transcription and processing during T cell development. J Immunol. 2012;188(7):3257–67. Epub 2012/03/02. doi: jimmunol.1103175 [pii] 10.4049/jimmunol.1103175 22379031 PMC3934760

[38] Fragoso R, Mao T, Wang S, Schaffert S, Gong X, Yue S, et al Modulating the strength and threshold of NOTCH oncogenic signals by mir-181a-1/b-1. PLoS Genet. 2012;8(8):e1002855 Epub 2012/08/24. 10.1371/journal.pgen.1002855 PGENETICS-D-12-00550 [pii]. 22916024 PMC3415433

[39] Henao-Mejia J, Williams A, Goff LA, Staron M, Licona-Limon P, Kaech SM, et al The microRNA miR-181 is a critical cellular metabolic rheostat essential for NKT cell ontogenesis and lymphocyte development and homeostasis. Immunity. 2013;38(5):984–97. Epub 2013/04/30. doi: S1074-7613(13)00191-X [pii] 10.1016/j.immuni.2013.02.021 23623381 PMC3738211

[40] Roark CL, Aydintug MK, Lewis J, Yin X, Lahn M, Hahn YS, et al Subset-specific, uniform activation among V gamma 6/V delta 1+ gamma delta T cells elicited by inflammation. J Leukoc Biol. 2004;75(1):68–75. Epub 2003/10/04. 10.1189/jlb.0703326 [pii]. . 14525969

[41] Gerber DJ, Azuara V, Levraud JP, Huang SY, Lembezat MP, Pereira P. IL-4-producing gamma delta T cells that express a very restricted TCR repertoire are preferentially localized in liver and spleen. J Immunol. 1999;163(6):3076–82. Epub 1999/09/08. doi: ji_v163n6p3076 [pii]. . 10477572

[42] Turchinovich G, Hayday AC. Skint-1 identifies a common molecular mechanism for the development of interferon-gamma-secreting versus interleukin-17-secreting gammadelta T cells. Immunity. 2011;35(1):59–68. Epub 2011/07/09. doi: S1074-7613(11)00264-0 [pii] 10.1016/j.immuni.2011.04.018 . 21737317

[43] Hamada S, Umemura M, Shiono T, Tanaka K, Yahagi A, Begum MD, et al IL-17A produced by gammadelta T cells plays a critical role in innate immunity against listeria monocytogenes infection in the liver. J Immunol. 2008;181(5):3456–63. Epub 2008/08/21. doi: 181/5/3456 [pii]. 18714018 10.4049/jimmunol.181.5.3456 PMC2859669

[44] Shibata K, Yamada H, Nakamura R, Sun X, Itsumi M, Yoshikai Y. Identification of CD25+ gamma delta T cells as fetal thymus-derived naturally occurring IL-17 producers. J Immunol. 2008;181(9):5940–7. Epub 2008/10/23. doi: 181/9/5940 [pii]. . 18941182 10.4049/jimmunol.181.9.5940

[45] Simonian PL, Roark CL, Wehrmann F, Lanham AM, Born WK, O'Brien RL, et al IL-17A-expressing T cells are essential for bacterial clearance in a murine model of hypersensitivity pneumonitis. J Immunol. 2009;182(10):6540–9. Epub 2009/05/06. doi: 182/10/6540 [pii] 10.4049/jimmunol.0900013 19414809 PMC2766088

[46] Shibata K, Yamada H, Sato T, Dejima T, Nakamura M, Ikawa T, et al Notch-Hes1 pathway is required for the development of IL-17-producing gammadelta T cells. Blood. 2011;118(3):586–93. Epub 2011/05/25. doi: blood-2011-02-334995 [pii] 10.1182/blood-2011-02-334995 . 21606479

[47] Shibata K, Yamada H, Nakamura M, Hatano S, Katsuragi Y, Kominami R, et al IFN-gamma-producing and IL-17-producing gammadelta T cells differentiate at distinct developmental stages in murine fetal thymus. J Immunol. 2014;192(5):2210–8. Epub 2014/02/04. doi: jimmunol.1302145 [pii] 10.4049/jimmunol.1302145 . 24489104

[48] Li J, Wu D, Jiang N, Zhuang Y. Combined deletion of Id2 and Id3 genes reveals multiple roles for E proteins in invariant NKT cell development and expansion. J Immunol. 2013;191(10):5052–64. Epub 2013/10/15. doi: jimmunol.1301252 [pii] 10.4049/jimmunol.1301252 24123680 PMC3837387

[49] Arase H, Ono S, Arase N, Park SY, Wakizaka K, Watanabe H, et al Developmental arrest of NK1.1+ T cell antigen receptor (TCR)-alpha/beta+ T cells and expansion of NK1.1+ TCR-gamma/delta+ T cell development in CD3 zeta-deficient mice. J Exp Med. 1995;182(3):891–5. Epub 1995/09/01. 7650493 10.1084/jem.182.3.891 PMC2192151

